# The Immunosuppressant Protosappanin A Promotes Dendritic Cell-Mediated Expansion of Alloantigen-Specific Tregs and Prolongs Allograft Survival in Rats

**DOI:** 10.1371/journal.pone.0066336

**Published:** 2013-06-26

**Authors:** Maomao Zhang, Shuo Zhang, Jian Wu, Yong Sun, Lili Li, Wenjuan Du, Jingjin Liu, Jingbo Hou, Bo Yu

**Affiliations:** 1 The Key Laboratory of Myocardial Ischemia, Chinese Ministry of Education, Harbin, Heilongjiang Province, China; 2 Department of Cardiology, Second Affiliated Hospital of Harbin Medical University, Harbin, Heilongjiang Province, China; Université Libre de Bruxelles, Belgium

## Abstract

Protosappanin A (PrA), an immunosuppressive ingredient of the medicinal herb *Caesalpinia sappan* L, prolongs heart allograft survival in rats, possibly by impairing the function of antigen-presenting cells (APCs). We examined the effects of PrA on the maturation and function of dendritic cells (DCs), a potent class of APCs, and the downstream cell–cell and intracellular signaling pathways mediating the immunosuppressive activity of PrA. PrA inhibited LPS-stimulated maturation of Wistar rat DCs in vitro as reflected by reduced expression of costimulatory molecules (CD80 and CD86) and reduced expression of TLR4 and NF-κB, two critical signaling components for antigen recognition. PrA also enhanced the release of IL-10 and decreased the release of IL-12 from DCs, but had no effect on the production of TGF-ß. In mixed cultures, Wistar DCs pretreated with PrA impaired the proliferation of Sprague Dawley (SD) rat T cells while promoting the expansion of SD rat CD4^+^CD25^+^ regulatory T cells (Tregs). Both oral PrA treatment and infusion of PrA-pretreated Wistar DCs prolonged cardiac allograft survival (Wistar donor, SD recipient) and expanded recipient CD4^+^CD25^+^Foxp3^+^ Tregs. Donor spleen cells, but not spleen cells from a third rat strain (DA), supported the expansion of recipient CD4^+^CD25^+^Foxp3^+^ Tregs and suppressed recipient T cell proliferation. We conclude that PrA triggers a tolerogenic state in DCs that allows for the induction of alloantigen-specific Tregs and the suppression of allograft rejection in vivo.

## Introduction

Protosappanin A (PrA), a potent immunosuppressive agent derived from the traditional medicinal herb *Caesalpinia sappan* L, prolonged the survival of heart allografts in rat and prevented immune-mediated tissue damage [1], [2]. Inhibition of acute tissue rejection was associated with reduced proliferation of recipient T cells and underexpression of NF-κB in infiltrating monocytes [3], but little is known of the intercellular and molecular signaling mechanisms for PrA-mediated immunosuppression.

An ideal clinical immunosuppsant for prevention of tissue rejection in transplant patients should impair only those antigen presenting cells (APCs) responsive to donor antigens [4]. Dendritic cells (DCs) are the most potent APCs and are thus a potential target for the induction of antigen-specific immunne tolerance. Indeed, DCs in an immunotolerant state expanded alloantigen-specific regulatory T cells (Tregs), while infusion of tolerogenic DCs prolonged allograft survival, likely by regulating the host T cell response [5]–[8].

In this study, we examined the effect of PrA on DC phenotype and compared the effects of native and PrA-treated DCs on the proliferation and phenotype of T cells to elucidate the signaling mechanisms of PrA-mediated immunosuppresion. PrA inhibited LPS-stimulated DC maturation, decreased DC TLR4/NF-κB expression and activity, induced T-cell hyporesponsiveness, and expanded Tregs in vitro. Furthermore, PrA administration or transfer of PrA-treated donor DCs led to recipient T cell hyporesponsiveness and Treg expansion as well as suppression of allograft rejection in a rat model of heart transplantation. This study identifies the probable mechanisms for the immunosuppressive effect of PrA.

## Methods

### Experimental Animals

Specific pathogen free (SPF) male Wistar (recipient, 180–220 g), Sprague Dawley (SD, donor, 200–250 g), and DA rats (180–220 g) were obtained from experimental animal center of Beijing for heart transplantation. The animals were maintained under standard conditions and fed rodent food and water according to the laboratory animal care principles and the guide for the care and use of laboratory animals in our institution.

### Drug preparation

The heartwood of *Caesalpinia sappan* L was supplied by San Keshu Chinese Medical Market (Harbin, China) and the identity confirmed by the Pharmacy Faculty of HeiLongjiang University of Chinese Medicine.

The active PrA was extracted as described [1]. In brief, heartwood was soaked three times in 95% ethanol and the PrA recovered from the ethanol by silica gel column chromatography. The isolated compound was identified by the specific wave spectrum and the purity shown to be more than 98%. PrA was then dissolved in sterile distilled water to different concentrations for experiments.

### Generation of bone marrow-derived DCs and PrA treatment

DCs were derived from the bone marrow precursors of Wistar rats as described previously [9]. Briefly, bone marrow was flushed from femurs and passed through a 100-mm pore mesh to remove fibrous tissue. The red blood cells were lysed. Then the cells were cultured at 10^6^ cells/ml in RPMI 1640 medium (HyClone, Logan, UT) supplemented with 10 ng/ml each of rat GM-CSF and IL-4 (PeproTech, Rocky Hill, NJ). To induce DC maturation, cells at day 6 in vitro were treated for 48 h with lipopolysaccharide (LPS, 10 ng/ml; Sigma-Aldrich, St. Louis, MO). The DC phenotype was confirmed by anti-OX62 labeling and flow cytometry using a FACS Calibur system (detailed below). Only those preparations with a purity >85% DCs were used for subsequent experiments. In other experiments, the DCs at day 6 were treated for 48 h with LPS plus different doses of PrA.

### Flow cytometry

DC purity and phenotype were determined by surface expression of specific markers using flow cytometry. The following conjugated monoclonal antibodies (mAbs) were used: FITC-OX62, PE-CD80, and PE-CD86 (eBioscience, San Diego, CA). For each staining protocol, the appropriate isotype-matched control was included.

For analysis of Treg expression phenotype, cells in mixed lymphocyte reactions (MLRs, prepared as described below) were washed twice with PBS and then stained for 30 min at 4°C with the following conjugated primary mAbs: CD4-FITC, CD25-allophycocyanin, and Foxp3-PE (all from eBioscience). After primary antibody incubation, the cells were washed, fixed, permeabilized using fixation/permeabilization reagents (supplied by eBioscience), and stained for intracellular Foxp3. All reagents were used at optimal concentrations as determined by pilot experiments. Analysis of cell phenotype and cell counting were performed using FACS Calibur and analyzed using CellQuest Pro software.

### Cell viability

Cell viability was assessed by the mitochondrial dehydrogenase-dependent reduction of the yellow tetrazolium dye MTT to insoluble purple formazan. Cells were seeded at 0.5×10^6^ cells/well in 96-well plates and treated with different doses of PrA (0, 5, 20, or 40 nM) for 48 h. Following PrA treatment, 0.2 ml of MTT stock solution (5 mg/ml, Sigma) was added to each well and the plates incubated for an additional 4 h at 37°C. The supernatant was removed and 100 µl of DMSO added to each well to dissolve the accumulated formazan. The absorbance at 570 nm, indicative of viable cell number, was measured on a microplate reader. The assays were performed on at least three independent cultures and each PrA concentration was tested in triplicate within each microwell plate.

### Mixed lymphocyte response (MLR) assay

Allogeneic T cells were isolated from SD rat spleens by non-adherence to nylon wool and cocultured at different plating ratios with Wistar DCs preactivated with LPS alone or LPS plus PrA to assess the impact of DC functional state on T cell proliferation and function. 1×10^6^ isolated SD rat T cells were cultured for 5 d in 96-well round-bottom microplates in the presence of Wistar DCs (control, LPS-treated, or LPS+PrA-treated; as stimulator) treated with the antimitotic agent mitomycin C (10 mg/ml) at different DC∶T cell ratios (1∶10, 1∶20, 1∶50, or 1∶100). These cocultures were incubated with 10 mM BrdU for 24 h, and BrdU incorporation by proliferating responder cells (T cells) was quantified using a BrdU ELISA according to the manufacturer's instructions (Chemicon International, Temecula, CA). All proliferation assays were performed in triplicate.

To study the immunosuppressive effect of Tregs on T cell proliferation, 1×10^5^ CD4^+^CD25^+^Tregs isolated from MLRs by magnetic-activated cell sorting (MACS, detailed below) were added to the coculture of mitomycin C-treated spleen cells and allogeneic T cells at a Treg/T cell/spleen cells ratio of 10∶10∶1. The proliferation of T cells was again detected by BrdU-ELISA.

### Cell sorting by MACS

The CD4^+^CD25^+^Tregs were isolated from cocultured MLRs by MACS [10], [11]. First, CD4^+^T cells were enriched by negative selection with anti-CD8 mouse IgG1 and anti-mouse IgG1 microbeads (Miltenyi Biotec). Subsequently, the total CD4^+^T cell population was incubated with anti-CD25 mouse IgG1 and anti-mouse IgG1 microbeads, then separated into CD4^+^CD25^+^ and CD4^+^CD25^−^ T cell fractions by positive selection using an MS column (Miltenyi Biotec). The purity of CD4^+^CD25^+^ T cells was consistently 80–90% as assessed by FACS (shown in Figure S1).

### Western blots

Western blotting was used to assess TLR4 and NF-κB expression in DCs. Briefly, cell lysates were obtained after exposure of DC cultures to LPS (with or without PrA) and blotted with anti-TLR4, anti-NF-κB p65, and anti-ß-actin (from Santa Cruz Biotechnology, Santa Cruz, CA). Target protein level was expressed as the densitometric ratio of target protein to ß-actin.

### Immunofluorescence staining

DCs grown on glass coverslips were fixed with 4% paraformaldehyde for 30 min at room temperature, blocked with goat serum, and incubated with primary Abs against TLR4 or NF-κB p65(1∶100) overnight at 4°C. After washing, cells were incubated with FITC-conjugated goat anti-mouse Ab (1∶200; Santa Cruz Biotechnology) for 1 h at 37°C. Nuclei were counterstained with DAPI (0.1 g/ml; Sigma-Aldrich). Fluorescent images were acquired with a confocal laser-scanning microscope (FluoView v5.0 FV300; Olympus, Tokyo, Japan).

### Electrophoretic mobility shift assay (EMSA)

Nuclear extracts of DCs were prepared with the nuclear extraction reagent (Pierce, Rockford, USA). Protein concentrations in the extracts were determined according to the Bradford assay (Bio-Rad, Hercules, CA, USA). To determine NF-κB activation, binding of NF-κB p65 was assessed in nuclear extracts by EMSA [12]. Double-stranded oligonucleotides for NF-κB p65 were labeled on the 3′-end with biotin by using the 3′-Biotin end-label kit from Pierce. EMSA was carried out by using the Lightshift kit from Pierce. Briefly, 10 µg of nuclear protein were incubated for 20 min at room temperature with a biotin-labelled oligonucleotide probe (5′-AGT TGA GGG GAC TTT CCC AGG C-3′for the NF-κB binding site). Protein DNA complexes were separated by using a 6% nondenaturing acrylamide gel electrophoresis. The reactions were transferred to a nylon membrane. The biotin-labeled DNA was detected with LightShift chemiluminescent electrophoretic mobility shift assay kit (Pierce).

### Heart transplantation and PrA treatment

Ectopic peritoneal heart transplantation was performed from Wistar to SD rats using the methods reported by Ono and Lindsey [13]. In brief, the ascending aorta and pulmonary artery of the donor heart were anastomosed bilaterally to the recipient abdominal aorta and inferior vena cava. Graft function was monitored by daily abdominal palpation and scored from 0 (no contractions) to 4 (vigorous contractions). Hearts were considered acutely rejected when the palpation score was <1. After heart transplantation, surviving animals were randomly divided into 3 groups. One group was given 25 mg/kg oral PrA every day from day 0 to 7 after transplantation, the second PrA-conditioned Wistar DCs at 2.5×10^6^ cells per rat on days 1, 3, and 5 postsurgery, and the third with the same volume of PBS. Animal procedures were approved by the institutional Animal Ethics Committee of Harbin Medical University.

### ELISA analysis

Cytokine levels in culture supernatant or rat serum were detected by two-site sandwich ELISA using antibody pairs against IL-12p70, IL-10 and TGF-ß (R&D Company, US). Samples were assayed in triplicate and were quantitated by comparison to standard curves generated using purified recombinant cytokines.

### Quantitative RT-PCR

Briefly, total RNA was harvested from the cells, reverse-transcribed to cDNA and amplified using SYBR Premix Ex Taq II (TaKaRa, Dalian, China) with primers. PCR amplification was performed for 30 cycles consisting of the following steps: 5 min at 94°C for denaturing, 30 s at the optimal temperatures specific to the primers used for annealing and 30 s at 72°C for extension. House-keeping gene of GAPDH was included as an internal control. All reactions were performed in triplicate. The expression of mRNA relative to GAPDH was determined using the 2^−ΔΔCt^ method.

The following primers were used in this study: IL-12, forward, 5′-TGATGATGACCCTGTGCCTT-3′ and reverse, 5′-GCATGGAGCAGGATACAGAGC-3′; IL-10, forward, 5′-CAGACCCACATGCTCCGAGA-3′ and reverse, 5′-CAAGGCTTGGCAACCCAAGTA-3′; TGF-ß, forward 5′- CTCAACACCTGCACAGCTCC-3′, reverse 5′-ACGATCATGTTGGACAACTGCT-3′; GAPDH, forward, 5′-TTCA TTGACCTCAACTAC-3′, reverse, 5′-AGACTCCACGACATACTC-3′.

### Statistical analyses

Data is expressed as mean ± standard error of the mean. Differences between nonparametric data sets were examined by the Mann-Whitney test. Multiple group means were compared by one-way ANOVA. Differences were considered statistically significant at P<0.05.

## Results

### PrA suppressed LPS-induced DC maturation

**Figure 1:**
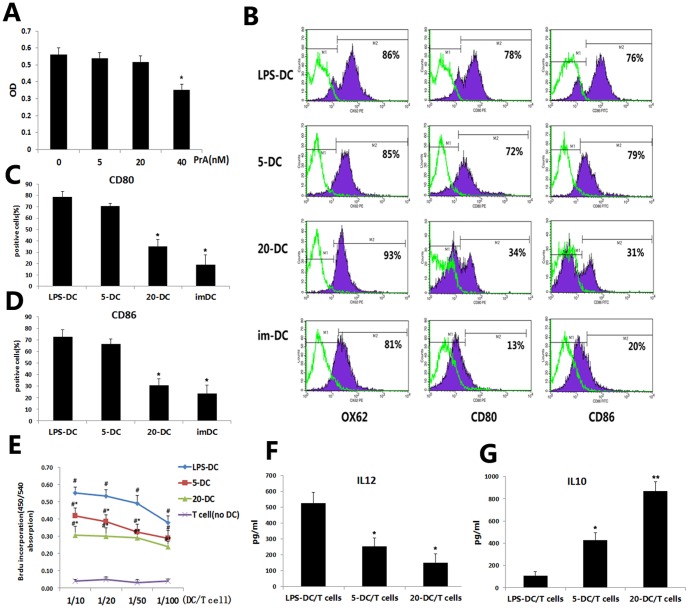
PrA inhibited DC maturation responding to LPS and diminished DC-mediated T cell allostimulation. DCs (Wistar) were treated with LPS alone or LPS plus PrA (5, 20, or 40 nM) from days 7 to 9 in vitro. (A) DC viability was measured at each concentration using the MTT assay. The highest dose induced a significant decrease in viable DC count, so only 5 nM and 20 nM were used in subsequent experiments. (B, C, D) LPS-induced maturation of DCs was examined by immunolabeling for OX62, CD80, and CD86 and subsequent FACS. Treatment with PrA suppressed the LPS-stimulated expression of these DC markers. (E) The proliferation of SD T cells in MLR coculture with mitomycin C-pretreated Wistar DCs (as stimulator) at DC∶T plating ratios of 1∶10, 1∶20, 1∶50, and 1∶100 was measured by Brdu incorporation. PrA-treated DC displayed much inferior T cell allostimulatory activity in MLR using 1×10^6^ SD T cells as responders. (F, G) IL-12p70 and IL-10 production in MLR coculture supernatant (DC and T cells ratio of 1∶10) were assessed by ELISA. Data are shown as the means±SD of three independent experiments (A, C–G; n = 3). Similar results were obtained in three independent experiments (B; n = 3). *P<0.05 and **P<0.01 compared with the LPS-DC (no PrA) control group. ^#^P<0.05, compared with the T cells only (no DC) group.

We first determined the non-toxic dose range of PrA for DCs in vitro using the MTT assay. Addition of PrA at 40 nM significantly decreased DC viability compared to control cultures (P<0.05) while lower doses did not (Fig. 1A). In subsequent experiments, 5 nM and 20 nM doses were used to examine the mechanisms of PrA-induced immunosuppression.

Immature DCs can be induced to mature by stimulation with LPS and is accompanied by increased surface expression of the co-stimulatory molecules CD80 and CD86. We investigated whether DCs treated with PrA still expressed these surface markers after activation by LPS. DCs cultured with the cytokines GM-CSF and IL-4 for 6 days were stimulated with LPS alone (10 ng/ml) or LPS plus PrA and cultured for an additional 48 h. After LPS stimulation, the levels of CD80 and CD86 were significantly higher in LPS-treated DCs (LPS-DC) compared with imDCs without LPS condition as indicated by flow cytometry. However cells pretreated with PrA (20 nM) plus LPS expressed markedly lower levels of CD80 and CD86 than DCs treated with LPS alone (LPS-DC). The difference of CD80 and CD86 expression between DCs treated with 20 nM PrA plus LPS (20-DC) group and imDC group was not statistical significant (Fig. 1B, C, D). The results suggested that PrA at a non-toxic dose (20 nM) impeded LPS-stimulated DC maturation as defined by surface CD80 and CD86 expression.

The effects of PrA on DC function were examined in cocultures containing Wistar-derived DCs and allogeneic T cells isolated from SD rat spleen (MLRs). Activated DCs normally induce allogeneic T cell proliferation; however, T cells from SD rats exhibited a reduced proliferative response in the presence of activated DCs pretreated with PrA compared to T cells cultured with PrA-naïve activated DCs (Fig. 1E). PrA-conditioned DCs not only depressed the allogeneic T cells proliferation, but also modulated the cytokines secretion in MLR cocultures with a higher IL-10 production and lower IL-12 production (Fig. 1F, G). Therefore, the capacity of LPS-stimulated DCs to undergo functional maturation was also inhibited by PrA.

### DCs pretreated with PrA enhanced the proliferation of Tregs in MLRs

**Figure 2:**
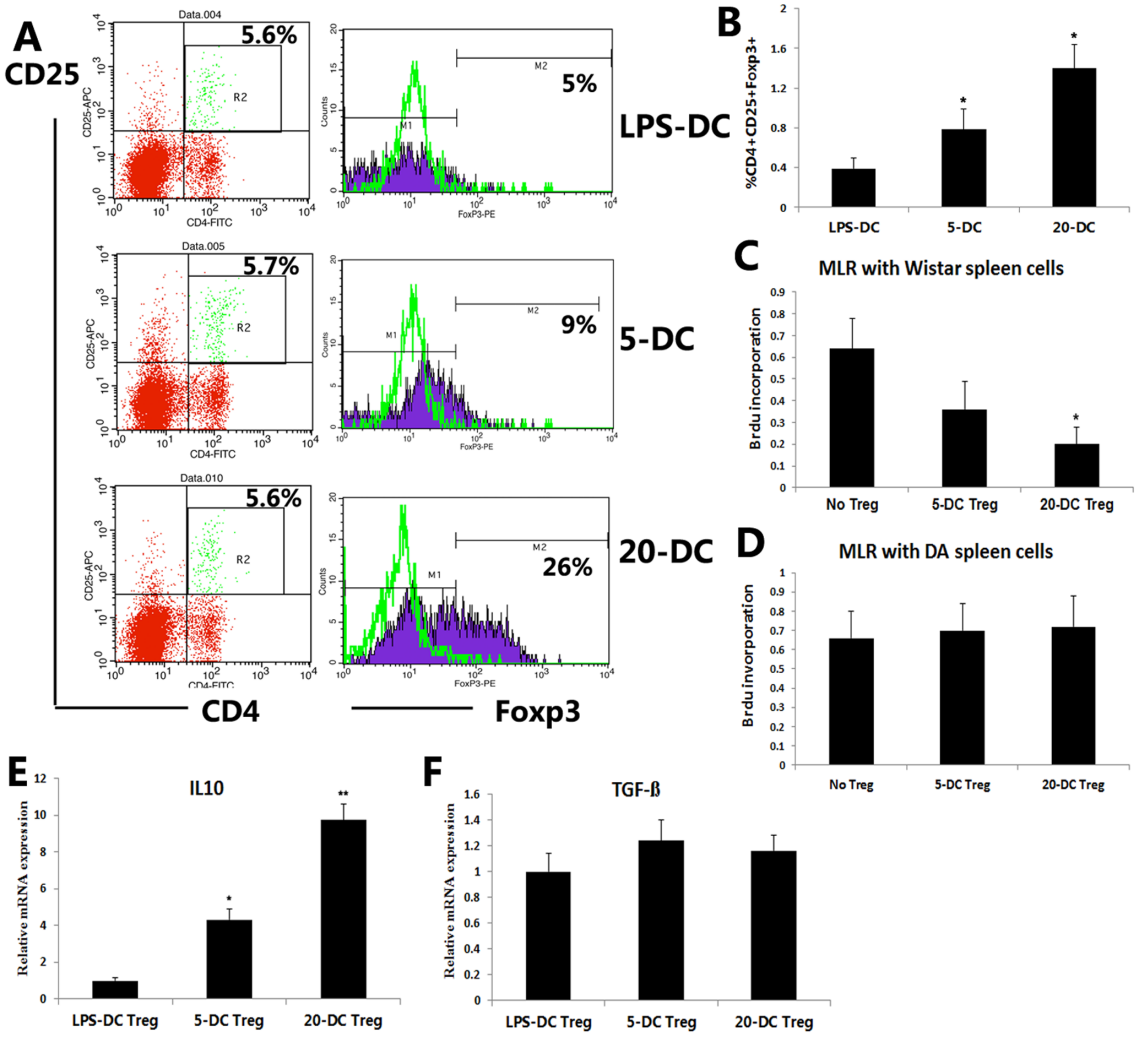
PrA-pretreated DCs induced the expansion of antigen-specific Tregs. (A, B) SD-derived T cells were cocultured with mitomycin C- pretreated Wistar DCs (with LPS or LPS plus PrA) for 2 days. Expression levels of the Treg markers CD4, CD25, and Foxp3 in coculture were measured by FACS. PrA-treated DC induced CD4^+^CD25^+^Foxp3^+^ Tregs expansion in a dose dependent manner. (C, D) SD-derived CD4^+^CD25^+^ Tregs were isolated from coculture with Wistar DCs by MACS and added to MLRs containing SD-derived T cells (responders). Either Wistar rat spleen cells or DA rat spleen cells (“third party” cells) were used as stimulator cells in these MLRs at a Treg∶T cell∶spleen cells ratio of 10∶10∶1. SD Tregs derived from 20 nM PrA-pretreated Wistar DC cocultures suppressed SD T cell proliferation (as evidence by reduced Brdu incorporation) compared to SD Tregs derived from PrA-naïve DC cocultures in the presence of Wistar spleen cells but not in the presence of DA (third party) spleen cells. (E, F) IL-10 and TGF-ß mRNA levels in these sortedTregs induced by DCs with or without PrA condition were assessed by qRT-PCR. Data are shown as the means±SD of three independent experiments (B–F; n = 3). Similar results were obtained in three independent experiments (A; n = 3). *P<0.05 compared to LPS-treated PrA-naïve DC controls.

To determine if Tregs were induced by PrA-treated DCs, the expression levels of the Treg surface markers CD4, CD25 and Foxp3 were examined by FACS in cocultures of Wistar DCs and SD-derived T cells. Tregs expressing CD4, CD25 and Foxp3 were significantly expanded in cocultures containing PrA-pretreated DCs compared to cocultures containing PrA-naïve DCs (Fig. 2A, B). We then assessed the effects of PrA-pretreated DCs on Treg function by measuring Treg immunosuppressive capacity on T cells. It is known that CD4^+^CD25^+^Tregs normally suppress T cell proliferation, so we separated CD4^+^CD25^+^Tregs from the mixture of DCs (PrA-treated or PrA-naïve) and T cells by MACS, and then cocultured these CD4^+^CD25^+^Tregs (suppressors) with SD-derived T cells (responders) and Wistar spleen cells treated with mitomycin C (stimulators) at a Treg∶T cell∶spleen cells ratio of 10∶10∶1. The proliferation of responder T cells by allogeneic spleen cells was suppressed by the addition of Tregs induced by PrA-pretreated DCs compared to Tregs induced by PrA-naïve DCs and the reduction in T cell proliferation was dependent on the PrA dose (Fig. 2C).

In order to determine if Tregs in MLRs mediated alloantigen-specific inhibition, DA rat spleen cells were used instead as a source of stimulator cells. In contrast to Wistar-derived stimulators, proliferation of SD-derived T cells in response to DA (“third party”) stimulator spleen cells was not affected by the addition of SD-derived Tregs induced by PrA-pretreated Wistar DCs (Fig. 2D). Tregs have been demonstrated to be able to produce both suppressive cytokines IL-10 and TGF-ß, so we measured IL-10 and TGF-ß mRNA expression in MACS-sorted CD4^+^CD25^+^Tregs to determine whether it is modulated by PrA. As shown in Fig. 2E–F, we found that only IL-10 mRNA level was significantly up-regulated in Tregs induced by the PrA-conditioned DCs, compared with Tregs induced by PrA naïve LPS-DCs assessed by qRT-PCR. The results indicated that the elevated IL-10 level, but not TGF-ß, may contribute to the functional capacity of Tregs primed by PrA-conditioned DCs and be implicated in the immunosuppressive mechanism of PrA. Collectively, our findings thus far suggest that PrA-conditioned DCs adopt an immunotolerant phenotype that supports the expansion of Tregs with antigen-specific immunosuppressive effects.

### PrA regulated the production of IL-10 and IL-12 by DCs in response to LPS

**Figure 3:**
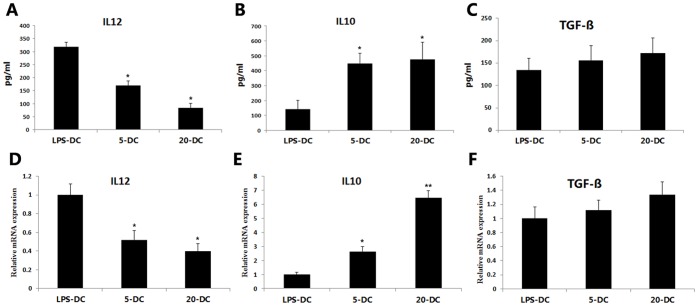
PrA modulated the IL-10 and IL-12 production of DCs responding to LPS. DCs were treated with LPS alone or LPS plus PrA (5 or 20 nM) from days 7 to 9 in vitro. The cytokines secretion and mRNA expression in DCs were assessed by ELISA and qRT-PCR. PrA suppressed IL-12p70 release (A) and mRNA level (D) from cultured Wistar DCs, accompanied with enhanced IL-10 release (B) and mRNA level (E). However PrA condition had no significant effect on TGF-ß production of DCs (C, F). The results are means ± SE of three independent experiments (n = 3). *P<0.05, **P<0.01 compared to LPS-DC controls.

Different cytokines act as pro-inflammatory or anti-inflammatory mediators, so the DC cytokine expression/secretion profile is one of the ultimate determinants of functional status. To investigate the effect of PrA on the cytokine secretion profile of DCs, the cytokine levels in culture supernatants and the mRNA levels in DCs were measured using ELISA assays and qRT-PCR. IL-10 and TGF-ß are key differentiation factors for the selection and effector function of Tregs; therefore, we compared the production of IL-10 and TGF-ß by PrA-treated and PrA-naïve DCs after LPS stimulation assessed by ELISA and qRT-PCR. Consistent with the immunotolerant phenotype induced by PrA, PrA-treated DCs produce more IL-10 secretion and mRNA expression than PrA-naïve DCs and this IL-10 production increased with the PrA dose (Fig. 3B, E). Concomitant with the increase in IL-10 secretion, we observed a PrA dose-dependent decrease in IL-12 release and mRNA expression from DCs (Fig. 3A, D). However neither secretion nor the mRNA level for TGF-ß showed a significant difference between the experimental groups (Fig. 3C, F), suggesting that TGF-ß might not be implicated in the immunosuppressive mechanism of PrA.

### PrA reduced the expression of TLR-4 and NF-κB by DCs

**Figure 4:**
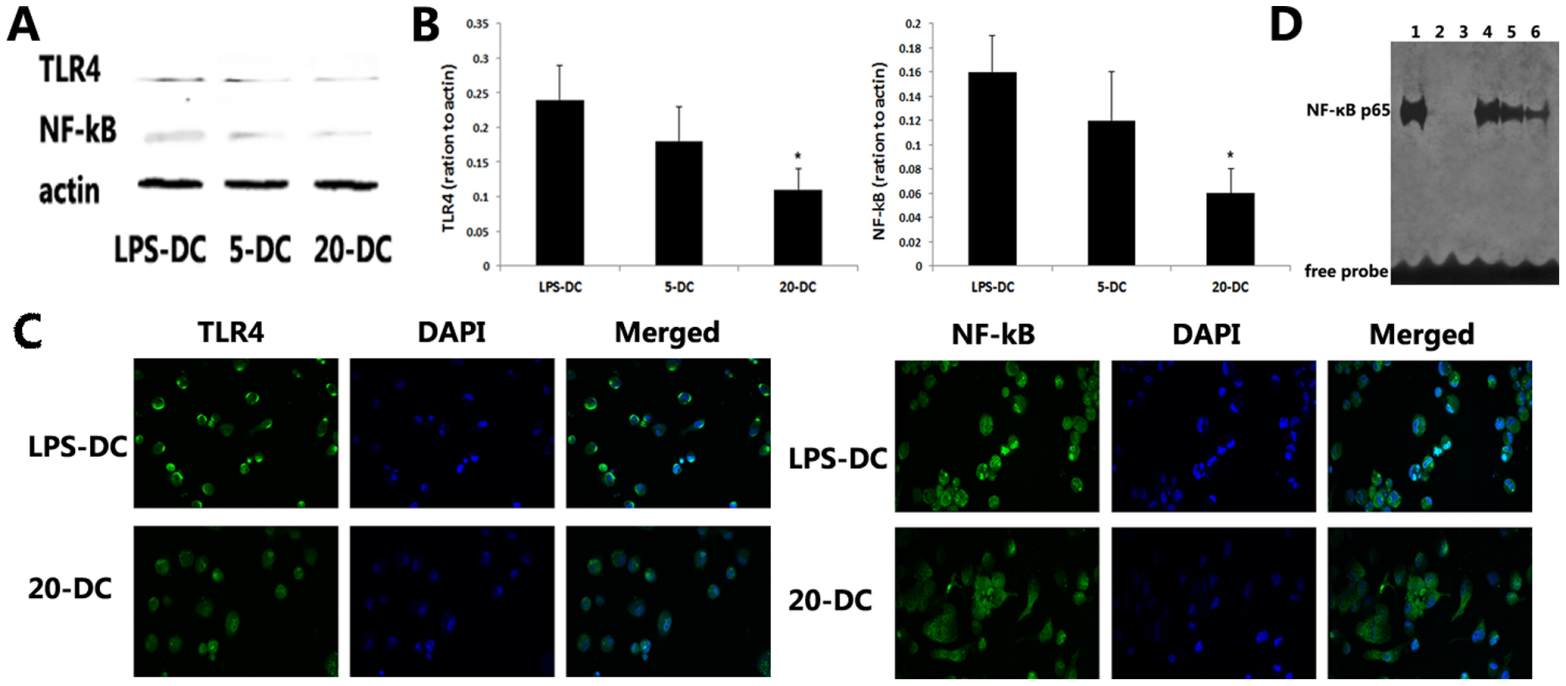
PrA suppressed TLR4 and NF-κB protein expression by DCs. DCs were exposed to PrA (0, 5, or 20 nM) and stimulated with LPS (10 ng/ml) for 48 h. TLR4 and NF-κB protein levels in DCs were detected by Western blot (A, B) and immunofluorescence (C). (D) NF-κB activity in DCs responding to LPS was depressed by PrA assessed by EMSA. Lane 1: positive nuclear extracts control containing active NF-κB; lane 2: negative nuclear extracts control without containing active NF-κB; lane 3: free oligonucleotide probe only; lane 4: LPS-DC; lane 5: 5-DC; lane 6: 20-DC. Data are shown as the means±SD of three independent experiments (B; n = 3). Similar results were obtained in three independent experiments (A, C, D; n = 3).*P<0.05 compared to LPS-DC controls.

The pattern recognition receptor family toll-like receptors (TLRs) contribute to antigen uptake, processing, and presentation. Toll-like receptor 4 (TLR4) plays a particularly crucial role in LPS-mediated DC activation. Expression of TLR4 and NF-κB by DCs was decreased in a dose-dependent manner by PrA as indicated by both Western blotting (Fig. 4A, B) and immunostaining of DCs (Fig. 4C). The NF-κB activation of DCs responding to LPS was also depressed by PrA condition as assessed by EMSA (Fig. 4D). Therefore, PrA might modulate DC function at least partially by depressing TLR4/NF-κB signaling.

### Injection of PrA-treated DCs promoted cardiac allograft survival

**Figure 5:**
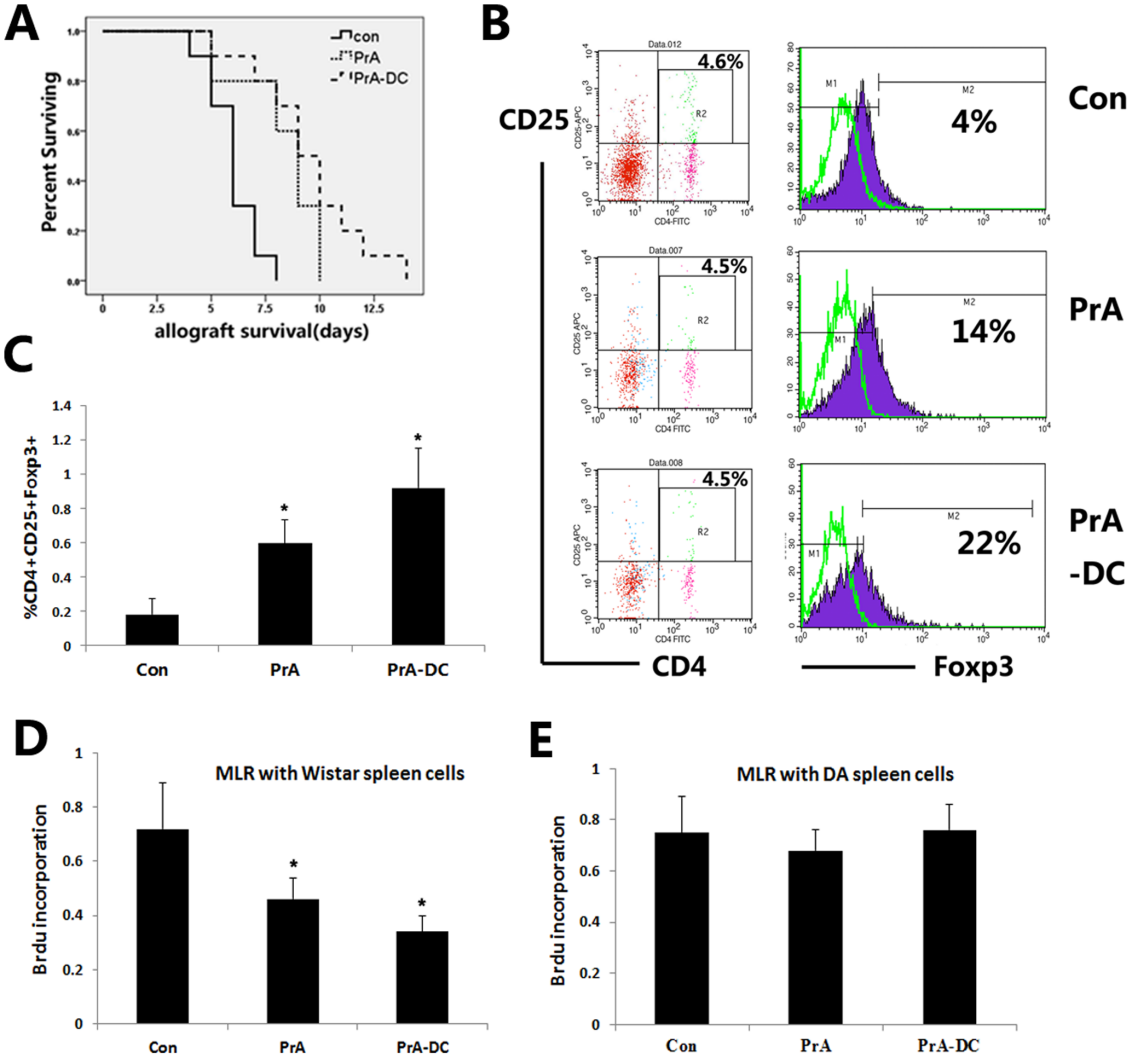
Both oral PrA and infusion of PrA-treated Wistar DCs prolonged allograft survival and expanded alloantigen-specific Tregs. (A) SD rats were transplanted with Wistar cardiac allograft and received different treatment posttransplantation. Infusion of PrA-treated DCs or oral PrA promoted cardiac allograft survival. Mean survival time was significantly longer for the PrA-treated DC group (9.000±0.791 days, n = 10) and PrA group (9.000±0.483 days, n = 10) compared to the PBS-injected control group (6.000±0.362 days, n = 10). (B, C) At day 7 posttransplantation, CD4^+^CD25^+^populations were gated in recipient spleen lymphocytes and Foxp3 expression was observed by FACS. Both PrA and PrA-treated DC infusion expanded the CD4^+^CD25^+^Foxp3^+^ Treg population in spleen cells of recipient rats. (D, E) Both PrA treatment and PrA-treated DC infusion suppressed T cell proliferation in an alloantigen specific manner in recipient rats. On day 7, T cells isolated from SD recipient rats (responders) were mixed with donor Wistar spleen cells or third party DA spleen cells in MLRs and T cell proliferation assessed by Brdu incorporation. The results are means ± SE of three independent experiments (C–E; n = 3). Similar results were obtained in three independent experiments (B; n = 3). *P<0.05 compared to LPS-treated PrA-naïve control DCs.

We previously demonstrated that PrA administration prolonged cardiac allograft survival and inhibited T cell proliferation, while the current in vitro studies revealed that PrA-pretreated DCs suppressed T cell proliferation, promoted Treg expansion, and enhanced the immunosuppressive capacity of Tregs on T cells. Thus, PrA may promote allograft survival through similar effects on immune effector cells in vivo. We first compared the survival of hearts from Wistar donors in SD recipients administered vehicle, PrA, or infused with PrA-conditioned Wistar DCs after transplant. Ten recipient rats were injected intravenously with 2.5×10^6^ PrA-treated DCs (n = 10) on days 1, 3, and 5 postsurgery while ten recipients were injected with the same volume of PBS (n = 10) on days 1, 3, and 5. The other ten rats were administered 25 mg·kg^−1^ PrA os every day (n = 10) from day 0 to 7 after transplantation. Both infusion of PrA-treated Wistar DCs and PrA administration prolonged graft survival compared to the PBS-injected controls (Fig. 5A).

### PrA induced the expansion of Tregs exhibiting alloantigen-specific immunosuppression in recipient rats

We investigated whether PrA induce the expansion of Tregs after transplantation by measuring CD4, CD25, and Foxp3 expression in the spleens of the three recipient groups. Consistent with in vitro results, recipient rats injected with PrA-treated DCs or treated with PrA exhibited a significant increase in the CD4^+^CD25^+^ Foxp3^+^ Treg population compared to recipient rats receiving only PBS (Fig. 5B, C).

To assess the immunosuppressive activity and antigen specificity of Tregs in recipient rats, we measured the proliferation of recipient (SD rat) T cells in the presence of mitomycin C-treated donor (Wistar rat) spleen cells or “third party” spleen cells from DA rats. Consistent with in vitro results, spleen cells from recipients administered either PrA or PrA-treated DCs showed reduced T cell proliferation compared to spleen cells from PBS-treated control recipients. In contrast, no reduction in T cell proliferation was observed when cocultured with third party spleen cells from DA rats treated with PrA or infused with PrA-treated DCs (Fig. 5D, E). These results strongly suggest that Tregs induced by PrA-treated DCs in vivo also exhibit donor-specific immunosuppression.

**Figure 6:**
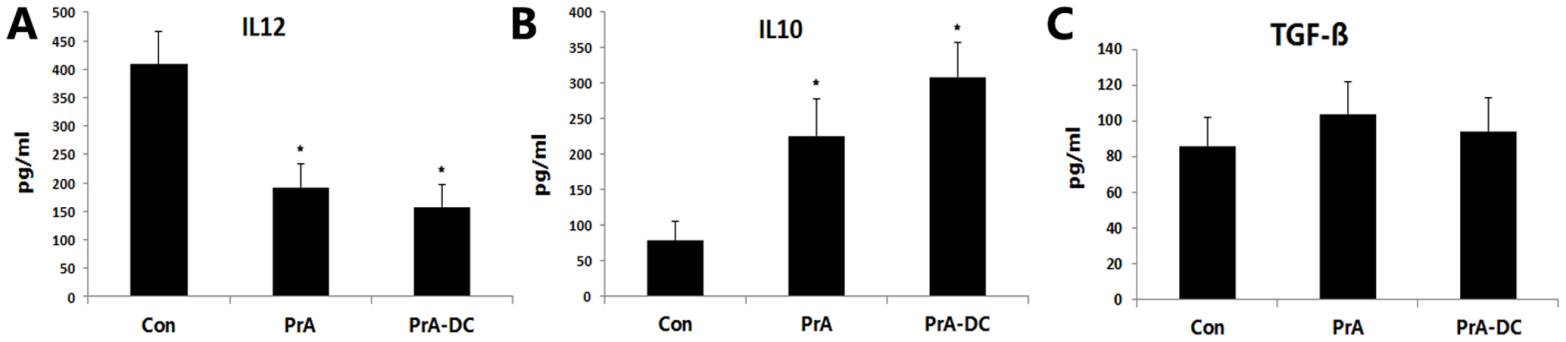
PrA modulated IL-10 and IL-12 production in vivo. Both PrA and PrA-treated DC infusion suppressed IL-12p70 production (A) and enhanced IL-10 production (B) in vivo as revealed by ELISAs of serum samples from recipient rats at day 7 posttransplantation. PrA showed no significant effect on TGF-ß production in serum of recipient rats (C). The results are means ± SE of three independent experiments (n = 3). *P<0.05 compared to the PBS-injected control group.

Finally, we also measured IL-10, IL-12 and TGF-ß concentrations in serum by ELISA and observed depressed IL-12 production (Fig. 6A) and increased IL-10 production (Fig. 6B) in the PrA-treated DC and PrA groups (both P<0.05) compared to the PBS control group, except for TGF-ß production (Fig. 6C). These findings were consistent with in vitro results and suggesting that PrA induces tolerogenic DCs in vivo.

## Discussion

Conventional immunosuppressants that inhibit T cell activity prevent rejection after transplantation. However, these agents impair both T cells targeting donor antigens and those that protect against microorganisms, increasing the risk of postoperative infection. An ideal immunosuppressant would impair only those APCs that respond specifically to donor antigens while not interfering with other immunological processes. Our previous studies demonstrated that PrA, an active component of *Caesalpinia sappan* L, promoted cardiac graft survival [1] and suppressed infiltrating T cell activity by inhibiting NF-κB activity [2]. In this study, we elucidated the cellular and molecular signaling pathways mediating this immunosuppressive effect. Of greater significance, we demonstrate that PrA acts to suppress immunity against donor antigens but not against third party antigens, suggesting that this compound may prevent post-transplantation rejection while maintaining immune protection against infection.

Dendritic cells are potent stimulators of naive T cells and key inducers of primary immune responses, including transplant immunity [4]. To identify the mechanisms of PrA-mediated immunosuppression, we examined the effects of this compound on DC maturation and of PrA-treated DCs on T cell and Treg functions. We demonstrate that PrA inhibits LPS-stimulated DC maturation, as evidence by the down-regulation of costimulatory molecules, and reduces the capacity of activated DC to stimulate T cell proliferation.

Dendritic cells can either activate T cells or induce T-cell tolerance depending on the state of maturation. Tolerogenic DCs in a CD80^−^CD86^−^ “immature” state reduce T cell proliferation and promote Treg differentiation [8], [14]–[17]. Indeed, the potential use of immature DCs as a cell-based therapy to promote immune tolerance following organ transplantation has been an area of active research, and transfer of immature DCs has been shown to act synergistically with pharmacotherapy to promote long-term allograft survival [7], [18]–[22]. In this study, we found that PrA induced a functional state that conferred the capacity to expand Tregs and induce an immunosuppressive (CD4^+^CD25^+^) Treg phenotype. Moreover, transfer of PrA-treated DCs promoted graft survival and induced Treg expansion in vivo, further confirming that PrA may act directly on DCs to enhance allograft immunity.

Regulatory T cells have crucial roles in maintaining tolerance and modulating adaptive immune responses. Accumulating evidence suggests that a favorable allograft outcome is associated with a robust population of CD4^+^CD25^+^Foxp3^+^ Tregs [23], [24]. This particular Treg phenotype effectively suppressed alloactivation of recipient T cells in response to donor antigens in MLR assays and the activation and expansion of antigen-specific cells [25], [26], thus contributing to allograft immunity [27]–[29]. In our study, PrA-conditioned DCs in the tolerant state not only induced T cell hyporesponsiveness, but also expanded Tregs exhibiting donor antigen-specific immunosuppression. Moreover, PrA administration or infusion of PrA-pretreated DCs impaired T cell responses and induced Tregs in the spleen after transplantation. These recipient Tregs, but not third party Tregs, induced T-cell hyporesponsiveness (i.e., an alloantigen-specific response).

In a previous study, we found that PrA inhibited NF-κB activation in grafts, resulting in reduced T cell proliferation in response to alloantigens [2]. Toll-like receptors (TLRs) play a major role in activating innate responses and directing adaptive immunity, including transplant immunity [30]–[32]. Toll-like receptor signals, especially signals activated by TLR4, lead to the rapid transcription of genes associated with inflammation. In APCs, TLR4 stimulation enhanced antigen presentation and up-regulated costimulatory molecules [33]. Several recent studies have examined the role of TLR agonists and TLR signals in allorecognition, rejection, and tolerance. Walker et al.reported that blockade of the TLR/MyD88/NF-κB pathway impaired DC function, rendering alloreactive T cells more susceptible to suppression by Tregs [34]. NF-κB has also been implicated in lymphocyte activation, proliferation, and survival of transplantation [30]. Consistent with our previous study, PrA suppressed TLR4 and NF-κB expression and activation in DCs and thus likely reduced downstream responses to alloantigens.

The tolerogenic effect of PrA on DCs was further confirmed by changes in cytokine production. IL-12 is an inflammatory cytokine, while IL-10 is a potent anti-inflammatory cytokine that suppresses alloimmunity and autoimmune responses [35], [36]. Furthermore, IL-10 is a key factor in the expansion and differentiation of Tregs and can enhance the long-term maintenance of allografts in vivo [37], [38]. PrA increased IL-10 production by DCs in a dose-dependent manner and sustained the function of donor rat heart in a transplant model. Thus, increased IL-10 release by DCs in response to PrA may mediate the induction of Tregs in vitro and in vivo. However another suppressive cytokine [39], TGF-ß production was not affected by PrA in DCs culture or serum of recipient rats, suggesting that TGF-ß was not involved in the immunosuppressive mechanism of PrA.

In conclusion, PrA may act by conferring a tolerogenic functional state on DCs that induces the expansion of alloantigen-specific Tregs, thereby reducing allograft immunity. We have identified the likely intracellular signals that mediate the immunosuppressive effect of PrA and provide strong support for the feasibility of PrA treatment as an immunotherapeutic strategy.

## Supporting Information

Figure S1 **Representative FACS result of sorted CD4^+^CD25^+^ T cells purity by MACS.**
(TIF)
