# Supplementary figures and images for: The Immunosuppressant Protosappanin A Promotes Dendritic Cell-Mediated Expansion of Alloantigen-Specific Tregs and Prolongs Allograft Survival in Rats
Source: PLoS One. 2013 Jun 26;8(6):e66336. doi: 10.1371/journal.pone.0066336 (PMC3694094; doi:10.1371/journal.pone.0066336)

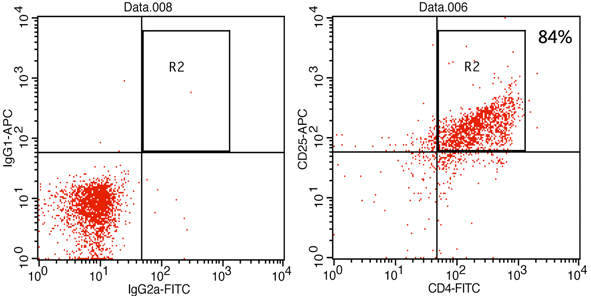

Supplement: Figure S1 — Representative FACS result of sorted CD4+CD25+ T cells purity by MACS. (TIF) [file pone.0066336.s001.tif]
